# Molecular and expression characterization of insulin-like signaling in development and metabolism of *Aedes albopictus*

**DOI:** 10.1186/s13071-023-05747-8

**Published:** 2023-04-18

**Authors:** Yi Dai, Xin Li, Jinying Ding, Zihan Liang, Renxian Guo, Tangwei Yi, Yihan Zhu, Siqi Chen, Shaohui Liang, Wenquan Liu

**Affiliations:** Department of Parasitology, School of Basic Medical Sciences, Wenzhou Medical University, Wenzhou, 325035 Zhejiang China

**Keywords:** *Aedes albopictus*, Insulin-like peptides, Insulin receptor, Metabolism, Development, dsRNA

## Abstract

**Background:**

Insulin-like signaling (IS) in insects is a conserved pathway that regulates development, reproduction and longevity. Insulin-like peptides (ILPs) activate the IS pathway by binding to the insulin receptor (InR) and trigger the ERK and AKT cascades. A varying number of ILPs were identified in *Aedes aegypti* mosquito and other insects. *Aedes albopictus* is an invasive mosquito which transmits dengue and Zika viruses worldwide. Until now, the molecular and expression characteristics of IS pathway in *Ae. albopictus* have not been investigated.

**Methods:**

The orthologues of ILP in *Ae. albopictus* genome assembly was analyzed by using sequence blast. Phylogenetic analysis and molecular characterization were performed to identify the functional domains of ILPs. Quantitative analysis was performed to determine the expression characteristics of ILPs, InR as well as ERK and AKT in mosquito development and different tissues of female adults after blood-feeding. In addition, the knockdown of InR was achieved by feeding larvae with *Escherichia coli*-producing dsRNA to investigate the impact of IS pathway on mosquito development.

**Results:**

We identified seven putative ILP genes in *Ae. albopictus* genome assembly, based on nucleotide similarity to the ILPs of *Ae. aegypti* and other insects. Bioinformatics and molecular analyses suggested that the ILPs contain the structural motif which is conserved in the insulin superfamily. Expression levels of ILPs, InR as well as ERK and AKT varied in *Ae. albopictus* development stages and between male and female adults. Quantitative analyses revealed that expression of ILP6, the putative orthologue of the insulin growth factor peptides, was highest in the midgut of female adults after blood-feeding. Knockdown of *Ae. albopictus* InR induces a significant decrease in the phosphorylation levels of ERK and AKT proteins and results in developmental delays and smaller body sizes.

**Conclusions:**

The IS pathway of *Ae. albopictus* mosquito contains ILP1-7, InR and ERK/AKT cascades, which exhibited different developmental and tissue expression characteristics. Feeding *Ae. albopictus* larvae with *E. coli*-producing InR dsRNA blocks the ERK and AKT cascades and interferes with the development of mosquito. Our data suggest that IS pathway plays an important role in the metabolism and developmental process and could represent a potential target for controlling mosquito-borne diseases.

**Graphical Abstract:**

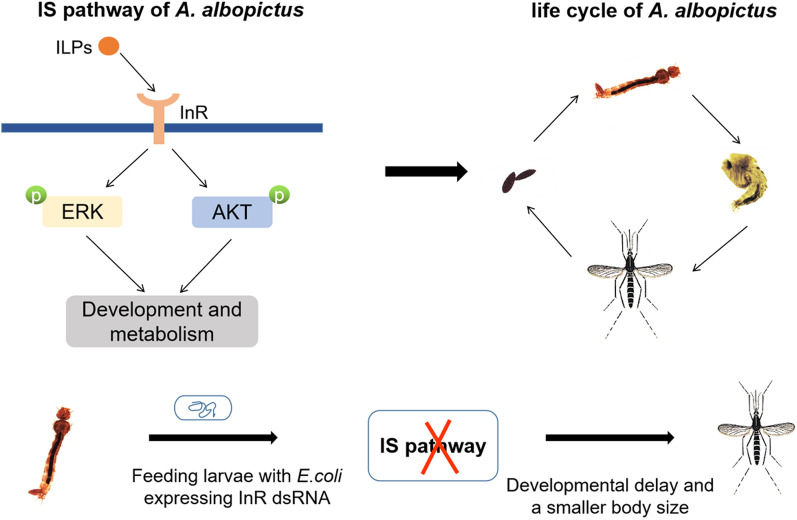

**Supplementary Information:**

The online version contains supplementary material available at 10.1186/s13071-023-05747-8.

## Background

Insulin-like signaling (IS) is a conserved pathway in all metazoan species and plays a critical role in regulating metabolism, growth, reproduction and lifespan [1, 2]. The IS pathway in insects is regulated by insulin superfamily peptide hormones, such as insulin-like peptides (ILPs) and insulin growth factor peptides (IGFPs) [2–4]. Most insect ILP/IGFLPs share a common structural motif called the insulin fold, which contains a signal peptide and B-chain, C-chain and A-peptides [2, 5]. The number of ILPs and IGFPs varies according to different insect species [4–6]. To date, eight ILPs in the genus *Drosophila* [4], five to eight ILPs in mosquitoes [5, 6] and 38 ILPs in *Bombyx mori* [7] have been identified.

The insect ILPs display differential expression characteristics in specific tissues and developmental stages [4–6]. For instance, in eight ILPs of *Drosophila*, the expressions of ILP1, -2, -3 and -5 are detected in the brains, while transcriptions of ILP4, -6 and -7 are found in the midgut, fat body and imaginal discs [4]. Similar spatiotemporal ILP expression patterns were also found in *Aedes aegypti* [5]. Transcripts of five ILPs of *Ae. aegypti* (ILP1, -3, -4, -7, -8) occurred predominantly in the heads (brains) of larval, pupal and adult mosquitoes; however, transcripts of ILP2, -5 and -6 were present not only in the head but also in the thorax and abdomens of *Ae. aegypti* [5]. Some ILP transcripts of *Anopheles* mosquitoes change in tissues from younger females relative to patterns observed in older females [6].

The IS pathway is initiated by the binding of ILPs to the insulin receptor (InR) [8, 9]. As a result of ILP stimulation, the InR undergoes tyrosine phosphorylation [8] and employs a group of adaptor molecules, known as insulin receptor substrates (IRSs), to initiate the phosphorylation of two main protein kinases: the extracellular regulated protein kinases (ERK) and the intracellular protein kinase B (also named AKT) [1, 10, 11]. The IS pathway regulates the nutrient metabolism processes and cell cycle responses by ERK/AKT cascades to affect development and reproduction [12] as well as host-pathogen interactions [13–15]. To date, a homologous receptor to the mammalian insulin receptor has been discovered in both flies and mosquitoes [4, 16]. Knockdown of InR blocks the IS pathway and results in growth defects, metabolic abnormalities and pathogen inhibition in several medical insects [17–20].

*Aedes albopictus*, also named “Asian tiger mosquito,” is the most invasive mosquito species which has been introduced to North and South America, Africa, Australia and Europe outside its original zones [21]. *Aedes albopictus* also transmits more than 25 arboviruses, including dengue, chikungunya and Zika virus [22]. Its fast adaptation to environments and the increasing resistance to common insecticides make the development of an efficient control strategy challenging [23]. In this study, we describe the expression characterization of the ILP gene family in *Ae. albopictus*. We identified seven putative ILP genes (ILP1–7) and one InR in *Ae. albopictus* genome assembly and characterized the expression features of ILPs and InR genes as well as ERK and AKT proteins during mosquito development and tissue of female adults following consumption of blood meals. We investigated the functional role of InR in *Ae. albopictus* mosquito development by feeding the larvae with dsRNA produced by the *Escherichia coli* expression system. Following the knockdown of InR in the larvae, we observed a significant delay in the development and a reduction in the wing size of *Ae. albopictus* adults. These results provide insights into the molecular characteristic of the IS pathway and its role during *Ae. albopictus* development and metabolism.

## Methods

### Sequence alignments and phylogenetic tree analysis

Putative ILP sequences from *Ae. albopictus* genome were investigated with the known *Ae. aegypti* ILP sequences using tblastn on the NCBI website (https://www.ncbi.nlm.nih.gov). ILP gene sequences of *Ae. aegypti, Anopheles gambiae* and *Drosophila melanogaster* were aligned with putative ILP genes of *Ae. albopictus* using ClustalX. The phylogenetic analysis was performed in the neighbor-joining method using the MEGA X phylogenetic software package. The signal peptides (SPs) were predicted processing sites by SignalP 5.0, and the amino acid alignments of ILPs were performed by using ESPript 3.0. The conserved amino acids in ILP motif and functional domains were identified by comparison to the sequences of the insulin superfamily published before [24]. The accession numbers of ILP mRNA sequences in NCBI are shown (Additional file 5: Table S1).

### Mosquito rearing, blood-feeding and tissue dissection

*Aedes albopictus* (Foshan strain) mosquitoes were maintained under the condition of 28 °C, 85% humidity with a 12-h light/dark photoperiod. The adult mosquitoes were provided with cotton pads soaked in a 10% sucrose solution. Larvae were fed with ground yeast. Female adults were starved for 16 h before blood-feeding. The blood was donated by healthy volunteers who provided written informed consent. Mosquitoes were allowed to feed the blood meals containing 1 mM ATP by using an artificial membrane feeder at 37 °C using a water jacket for 2 h. Only fully engorged mosquitoes were used for the experiment.

Four developmental stages of mosquitoes, eggs, larvae (L1 to L4 stages), pupae and adults (female and male), were collected to examine the temporal expression of IS pathway. Mosquitoe samples were prepared as described before [13]. In brief, mosquitoes were cold anesthetized, surfaced sterile in 75% ethanol and washed twice with sterile H_2_O. The different tissues of female adults including the head, thorax, fat body, midgut and ovary were separately dissected on ice. Samples in each treatment with 10–20 individuals were placed into 1.5-ml sterile Eppendorf tube containing 250 µl PBS buffer and homogenized in ice for 1 min. Then, the tissue homogenate was centrifuged at 12,000*g* for 5 min at 4 °C, and the supernatant was transferred to a new tube for RNA extraction.

### RNA extraction and gene expression analyses

Total RNA was isolated from the tissue homogenate using TRIzol reagent (Invitrogen) and treated with DNase. The cDNA was generated from total RNA using HiScript II Q Select RT Super-Mix (Vazyme Biotech, Nanjing, China) according to the manufacturer's instructions. Approximately 500 ng of total cDNA was employed for gene expression analyses by using the quantitative PCR (qPCR) method.

ChamQ SYBR qPCR Master Mix (Vazyme Biotech, Nanjing, China) was used for qPCR analysis with ABI QuantStudio 6 Pro Real-Time PCR System (Applied Biosystems, CA). The specific primers for *Ae. albopictus* ILP1-7 and InR genes are listed (Additional file 5: Table S2). The ribosomal S7 (RPS7) gene was used as the reference gene. Relative gene expression in different developmental stages was assessed by normalization to the levels of the RPS7 gene (2^−ΔCt^). The comparative Ct (2^−ΔΔCt^) method was used to calculate the relative expression of ILPs and InR genes in different tissues of blood-feeding female adults compared to the sugar-feeding adults.

### Western blotting analysis

Mosquito homogenate samples were lysed in 150 μl RIPA lysis buffer with phosphatase and protease inhibitors. After centrifugation of 12,000*g* for 10 min, the supernatants were collected, and the protein concentrations were detected by using a BCA protein assay kit. Total protein samples were separated on 12% SDS-PAGE polyacrylamide gels and transferred to a polyvinyl difluoride (PVDF) membrane. After blocking with 5% milk in Tris-buffered solution Tween-20 (TBS-T), the PVDF membrane was labeled with primary antibodies, which are separately used for detecting the total and phosphorylation of ERK (1:1000) as well as total and phosphorylation of AKT (1:1000) overnight at 4 °C. Subsequently, secondary antibodies of HRP-conjugated goat anti-rabbit IgG (1:2000) were added and incubated with each membrane for 1 h at room temperature. The signals were measured with Supersignal West Pico Chemiluminescent Reagent (Pierce, USA) for 5 min. The densitometric analysis of the phosphorylated-ERK and phosphorylated-AKT levels, as well as total ERK and total AKT levels, were performed by using the ImageJ software. The levels of phosphorylated and total ERK and AKT were separately normalized to that of β-actin. The fold change was indicated as the level of induction in each treatment compared with the control.

### Vector construction and dsRNA expression

Primers were designed according to *Ae. albopictus* InR gene (GenBank No. XM_029853782.1), which is an orthologous gene of *Ae. aegypti* InR [16]. The cDNA reverse transcribed from the total RNA of *Ae. albopictus* female adults was used as the template for PCR amplification. The fragment of the InR gene (from 3120 to 3530 nt in the CDS) was amplified by using PCR with primers InR-F (5′-GCTCTAGAGGTTCGCCTGGTGGACATTGAG-3′) and InR-R (5′-CCCTCGAGATCGTGGTGCCCATCGAGTCAT-3′). The InR gene with a length of 411 bp was inserted into the multiple clone sites of L4440 plasmid, which were flanked by two T7 promoters in an inverted orientation by using Xba I and Xho I restriction sites. The L4440-EGFP plasmid containing a 717-bp fragment targeting EGFP was used as the control. The L4440-InR and L4440-EGFP plasmids were confirmed by sequencing. Then the recombinant plasmids transformed into *E. coli* HT115 (DE3) competent cells lacking RNase III by a standard transformation procedure. The positive colonies were selected by using LB agar plate (100 μg/ml ampicillin) screen, PCR verification and DNA sequencing.

To produce dsRNA, a single colony of transformed bacteria was cultured in LB medium (100 μg/ml ampicillin) at 37 °C with shaking overnight. Then, 500 μl bacteria culture was added to 50 ml LB medium with antibiotics as above and incubated at 37 °C for 3–4 h (OD_600_ = 0.6). Expression of dsRNA was induced by adding 0.5 mM of isopropyl-β-d-1-thiogalactopyranoside (IPTG) for another 3 h.

### Feeding mosquito larvae with *E. coli* expressing dsRNA

Mosquito larvae feeding experiments were carried out by using recombinant bacteria expressing dsRNA as described before [25]. In brief, age-synchronized larvae (L3 stages) were placed into 10-cm dishes containing about 10 ml deionized water. The recombinant HT115 (DE3) *E. coli* was induced to generate dsRNA by adding IPTG as described above. The bacteria were pelleted by centrifugation (11,000*g*) for 5 min and resuspended in sterile water. Then, cells were mixed with 1% LB-agar containing IPTG and 1 g of finely ground yeast at 42 °C to keep the bacteria alive. The bacteria agar mixture was cubed into 5-mm cubes and fed to mosquito larvae once a day. Mosquito larvae were divided into three groups: InR dsRNA (dsInR) feeding group, EGFP dsRNA (dsEGFP) feeding group and the negative control (NC) groups fed with no bacterial LB mixture. Each group contained 10–20 larvae. After 2 days, the bacterial diet was removed and the normal diet was added to rear the larvae for developing into the pupae and adults. The transcript level of InR and phosphorylated level of ERK and AKT protein were detected as mentioned above. The visible phenotypic changes including the body sizes, pupation and eclosion times during the experiment were recorded daily for 2 weeks. Each treatment was repeated at least three times.

### Statistical analyses

Statistical analyses comparing gene expression were performed by using multiple comparison t-test [26]. Values of gene expression were represented as mean ± SD in at least three independent experiments. All statistical analyses were performed using GraphPad Prism8 software (GraphPad Software, Inc., La Jolla, CA): *P < 0.05, **P < 0.01, ***P < 0.005 and ****P < 0.001.

## Results

### Molecular and phylogenetic characterization of *Ae. albopictus* ILP genes

**Fig. 1:**
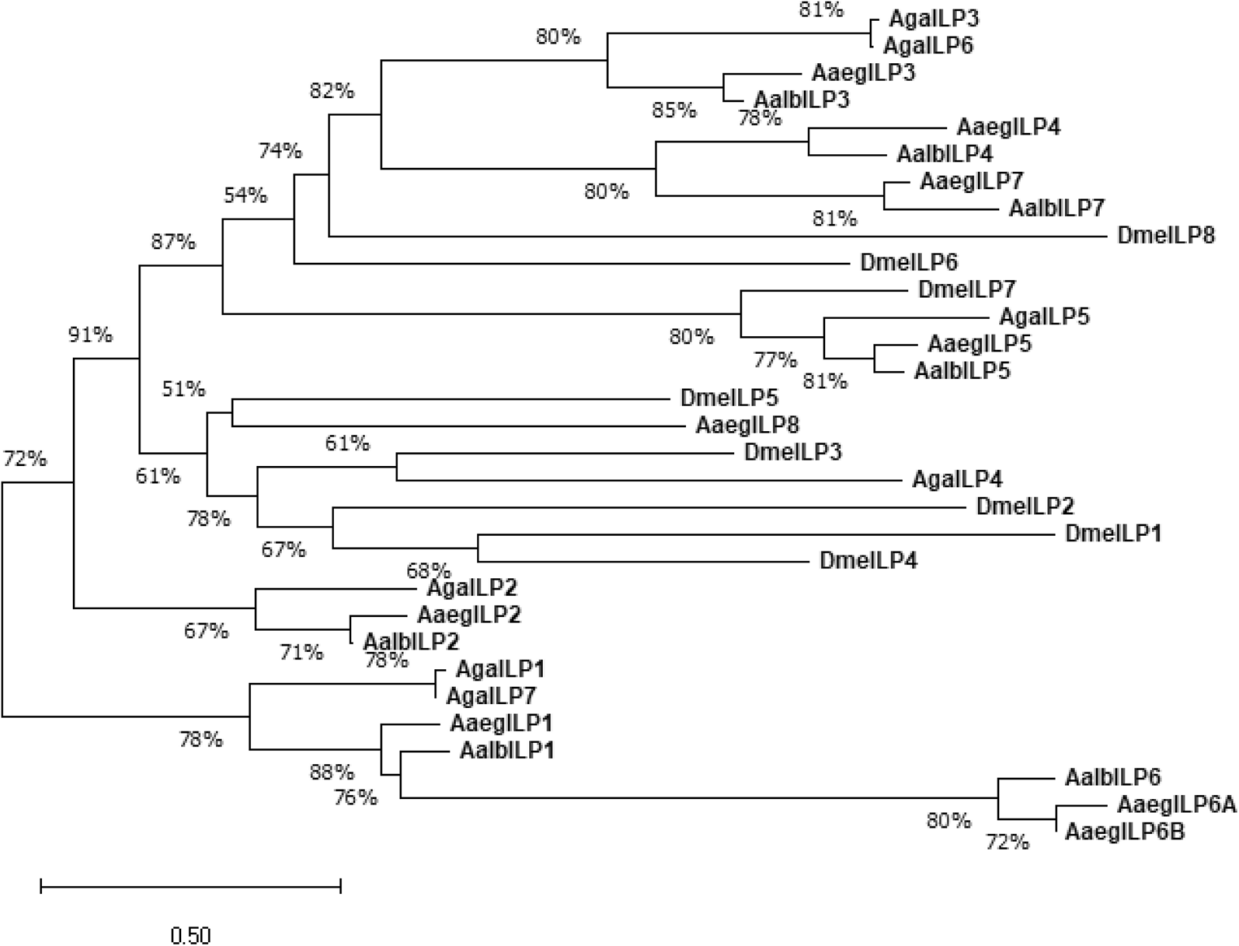
Phylogenetic analysis of *Aedes albopictus* mosquito ILPs by using the neighbor-joining method. The unrooted tree represented the main evolutionary relationships between ILP genes of *Ae. albopictus* (Aalb) and their orthologues in *Ae. aegypti* (Aaeg), *An. gambiae* (Aga) and *D. melanogaster* (Dme). Bootstrap values of 1000 replicates (%) are indicated on the branch nodes and represent the percentage of times that grouping was supported

Seven putative ILP transcripts in *Ae. albopictus* genomic assembly were obtained by using sequence blast with known *Ae. aegypti* ILP1-8 genes [5]. The phylogenetic analysis was performed to discern the evolutionary relatedness of *Ae. albopictus* ILPs with those identified in other insects, including *Ae. aegypti*, *An. gambiae* and *D. melanogaster*. We observed four clusters in the phylogenetic trees of ILPs, in which ILP3, -4, -5 and 7 are highly conserved in both *Ae. albopictus* and *Ae. aegpyti*. They are classified into a cluster with ILP3, -5 and -6 in *An. gambiae* as well as ILP6, -7 and -8 in *D. melanogaster* (Fig. 1). Another cluster is formed between ILP8 in *Ae. aegpyti* and ILP4 in *An. gambiae*, as well as ILP1-5 in *D. melanogaster*. The third cluster strongly supports the evolutionary relationship between ILP1and ILP6 in both *Ae. albopictus* and *Ae. aegpyti* as well as ILP7 in *An. gambiae*. Interestingly, mosquito ILP2 formed an independent branch separated from the other three clusters (Fig. 1). The bootstrap analysis revealed that the lineages of ILPs are highly conserved in *Aedes* mosquito species, but are variant with *D. melanogaster*. Additionally, the phylogenetic trees also suggested the evolutionary relatedness of InR among different insect species (Additional file 1: Fig. S1).

**Fig. 2:**
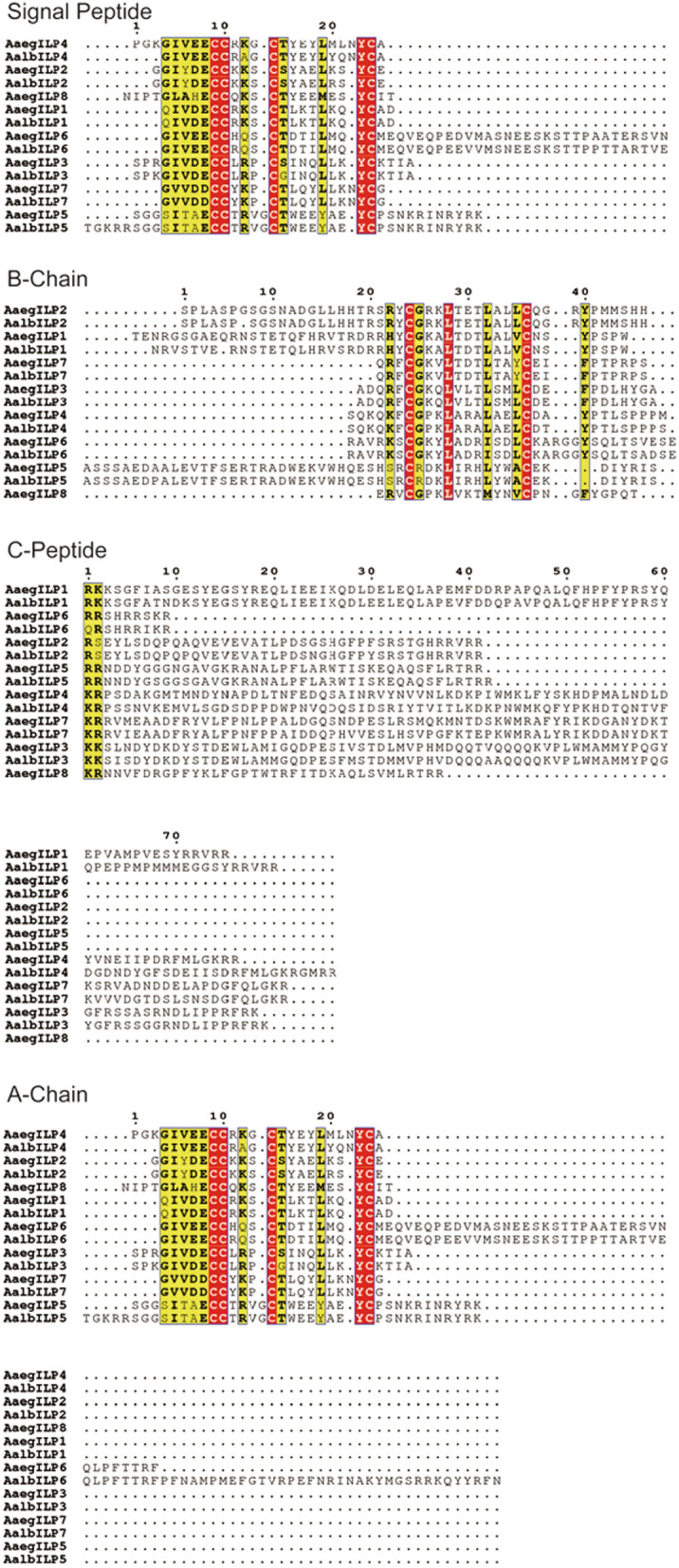
Amino acid sequence alignment of *Aedes* mosquito ILPs. Red highlights indicate all identical residues; yellow highlights indicate the majority conserved residues (black bold text indicates conserved residues). Alignment of ILPs in *Ae. albopictus* (Aalb) and *Ae.aegypti* (Aaeg) were performed by using ESPript 3.0 software

The conserved domain of ILPs in *Ae. albopictus* and *Ae. aegpyti* was analyzed by using amino acid sequence alignment. The open reading frames (ORFs) of ILP1-7 in *Ae. albopictus* encode the prepropeptides with 139–178 amino acid (aa) residues. The prepropeptides of ILPs contain a signal peptide (SP) (11 to 32 aa), B-chain, C-peptide and A-chain (Fig. 2). ILP1, -2 and -5 have extensions of 20–25 amino acids in the N-terminal of B-chain, which may generate an additional bioactive peptide by the proteolytic cleavage sites in Arg and Lys residues [5]. The length of C-peptide ranges from 9 to 81 residues and contains single or paired Arg and Lys amino acids, which may be additional proteolytic cleavage sites. After proteolytic cleavage of the SP and C-peptide, the ILP peptides form the tertiary structure by linkage of the two conserved Cys residues in the B-chain and four Cys residues in the A-chain. Short C-peptide and C-terminal extension sequences in ILP6 were found for both *Ae. albopictus* and *Ae. aegpyti*, which is considered the characteristic of IGFLP [27]. Both ILP4 and -7 possess an additional amino acid in the A-chain, which results in a nine-amino-acid spacer between the final two Cys residues instead of the usual eight—a feature not found in any other ILPs to date. ILP5 has an additional amino acid between the second and third Cys residues in the A-chain. No orthologues of *Ae. aegpyti* ILP8 were found in *Ae. albopictus*. Most members of ILPs in *Ae. albopictus* and *Ae. aegpyti* share identical or similar amino acids in the key position, which is conserved in the insulin superfamily.

### Expression of IS pathway genes during *Ae. albopictus* development

To explore the expression patterns of IS pathway-related genes in different developmental stages, samples from eggs, larvae, pupae and adults (male and female) were collected, respectively. A comprehensive survey of mRNA transcription and protein expression was conducted to investigate the temporal expression pattern of ILPs, InR as well as ERK and AKT proteins.

**Fig. 3:**
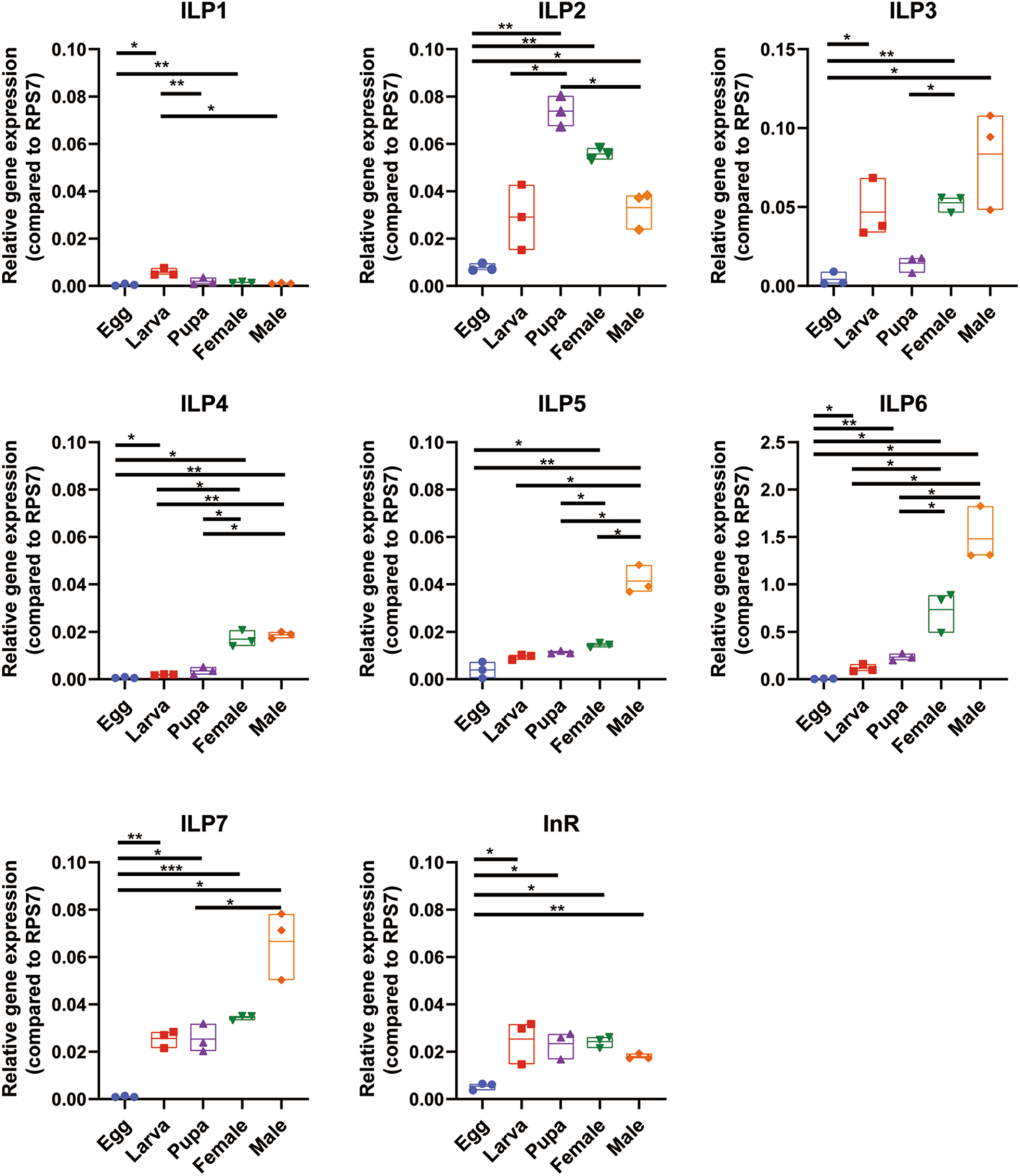
Expression of ILPs and InR in *Aedes albopictus* mosquito developmental stages and sexes. RNA was prepared from five specimens of egg, fourth-instar larvae, pupae, sugar-feeding females and males (all adult mosquitoes were collected at the same day time; 3 days post eclosion). Samples were subjected to RT-qPCR to assess relative expression of ILP1-7 and InR. RNA levels between samples were normalized to the RPS7 gene using 2^−ΔCt^ method. The midline indicates the median of the relative gene expression value. Data represent three biological replicates with 10–20 individuals in each treatment and are shown as mean ± SEM, *P < 0.05, **P < 0.01, ***P < 0.005, and representative results from a single experiment are shown

Relative expression analyses revealed ILP1-7 in the embryo stage exhibited much lower expression than those of post-embryonic development stages: larva, pupa and adult. The highest expression level of ILP1 was observed in the larva stage and then decreased in both pupa and adult stages (Fig. 3). ILP2 expression was detected at the highest level in the pupa stage and exhibited no significant difference between the male and female adults. We also detected an increased expression for ILP3, -4, -5, -6 and -7 in the adult stage compared with those in both larva and pupa stages. Most ILP expression showed no significant difference between the male and female adults except for ILP5, which exhibited a higher expression level for males. Meanwhile, we observed that InR exhibited higher expression in the postembryonic stages than those in the egg stage (Fig. 3).

**Fig. 4:**
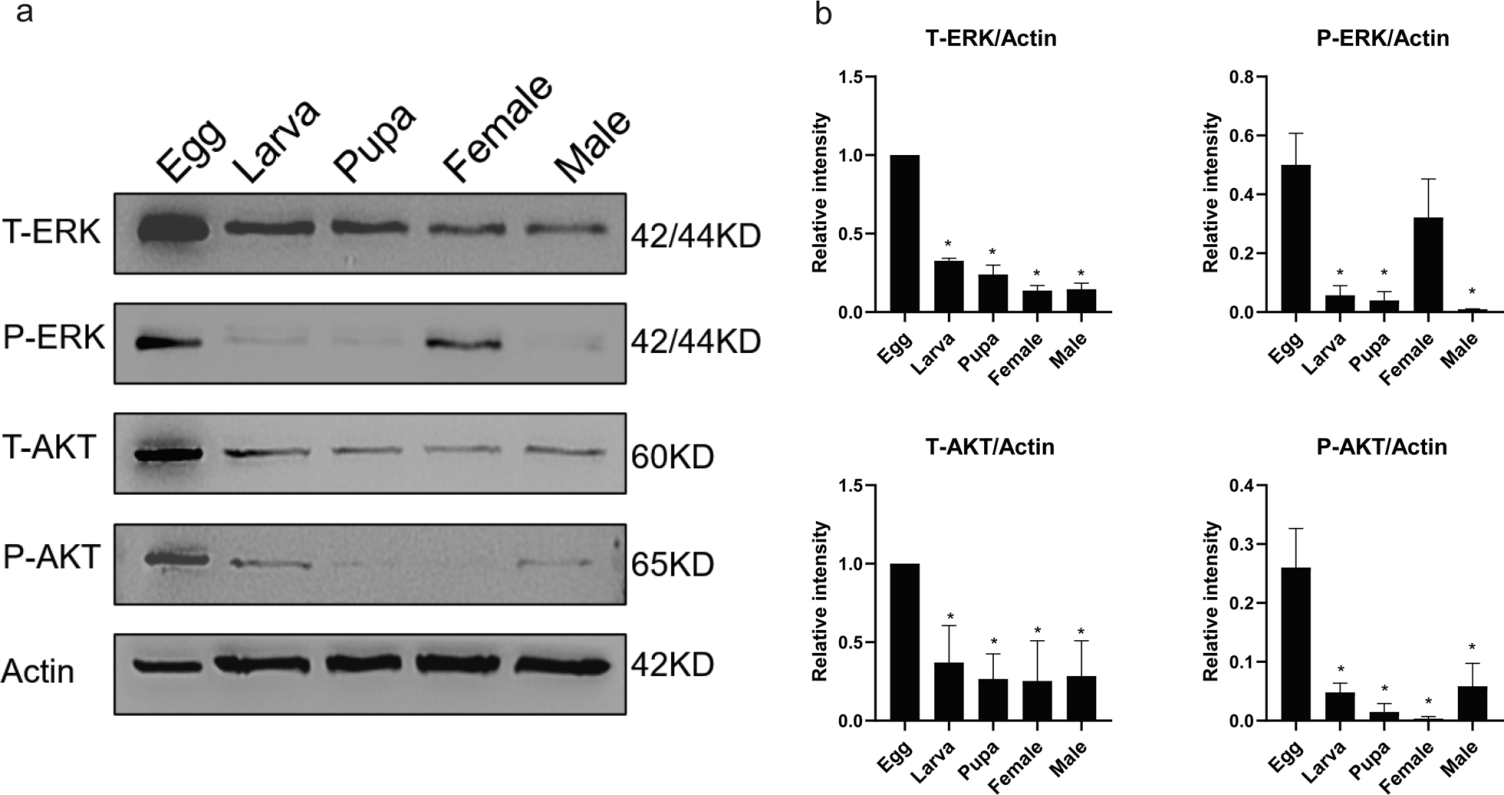
Expression analysis of ERK and AKT proteins in *Aedes albopictus* mosquito life stages. **a** The proteins of mosquito developmental stages: egg, larva, pupa and adults (female and male) were extracted and subjected to SDS-PAGE. Total and phosphorylation of ERK and AKT proteins were detected by Western blotting analysis. The ratios of ERK to actin were determined by using band density analysis. **b** The gray value analysis of total ERK/actin (T-ERK/actin), phosphorylated-ERK/actin (P-ERK/actin), total AKT/actin (T-AKT/actin) and phosphorylated-AKT/actin (P-AKT/actin). Data are presented as mean ± SEM from three independent experiments, *P < 0.05

Since the mRNA transcriptions of ILPs and InR are variants in *Ae. albopictus* development, we aim to examine whether two critical protein kinases, ERK and AKT, in the IS pathway also exhibit different expression patterns. As shown in Fig. 4, the protein levels of total ERK (T-ERK) and the phosphorylated ERK (P-ERK) were decreased in the larva, pupa and adult stages compared with those in the egg stage. Similarly, the total AKT (T-AKT) and phosphorylated AKT (P-AKT) protein levels were also downregulated when mosquito eggs developed into the postembryonic stages. Interestingly, we found the P-ERK protein level in the female adults is much higher than those in the male adults. In contrast, the P-AKT protein level in the male mosquito is much higher compared to those in the female mosquito (Fig. 4a and b). It suggested ERK/AKT cascades may be involved in regulating the different nutrition metabolisms in the male and the female mosquitoes.

### Impact of blood-feeding on the expression of IS pathway genes in *Ae. albopictus*

Relative expression analyses were applied to examine the tissue localization of seven ILP transcripts in female adults (Additional file 2: Fig. S2). We observed the higher transcription level for ILP3 in the head; ILP6 in the thorax, fat body and midgut; as well as ILP2 and 6 in the ovary. ILP1 transcript is rare in all tissues of female adults. InR transcript was high in both midgut and ovary. This revealed a differential distribution of ILP transcripts in female mosquito tissues.

**Fig. 5:**
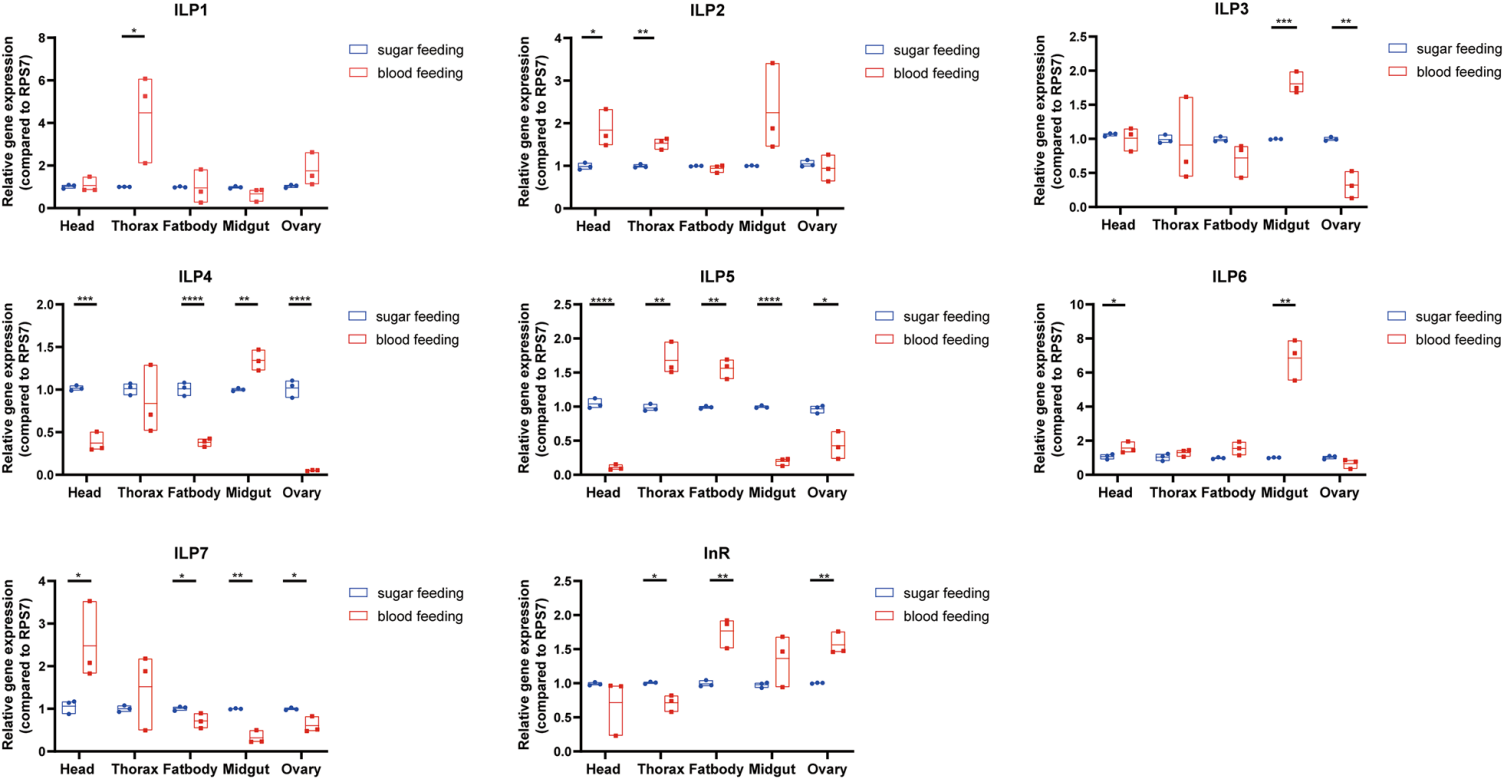
Expression of ILPs and InR in female mosquito tissues post sugar- feeding and blood-feeding. Relative expression levels of ILPs and InR genes in the head, thorax, fat body, midgut and ovary in sugar-feeding and 24 h post blood-feeding groups. RNA levels between samples were normalized to the RPS7 gene using 2^−ΔΔCt^ method. Data represent three biological replicates with 10–20 individuals in each and are shown as mean ± SEM. *P < 0.05, **P < 0.01, ***P < 0.005, ****P < 0.001

Female mosquitoes need to take the blood meal for developing eggs. To explore whether expression patterns of ILPs are affected by the diet switch from sugar to blood, we collected female adults after sugar and blood-feeding individually and investigated the expression patterns of ILPs in the mosquito head and peripheral tissues. Blood-feeding induced the upregulated expression for ILP2, -6 and -7 genes and the downregulated expression for ILP4 and -5 genes in the head. Meanwhile, the expression levels of ILP1, -2 and -5 genes in the thorax were increased after blood-feeding. The midgut is the main organ for blood digestion for mosquitoes. We found the expression levels of ILP3, -4 and -6 in the blood-feeding midguts were significantly increased compared to those in the sugar-feeding midguts (Fig. 5). In the mosquito fat body, the blood meals induced upregulated expression for ILP5 and downregulated expression for ILP4 and -7. The expression levels of ILP3, -4, -5 and -7 were decreased in the ovary after blood-feeding. In addition, we observed that the InR expression was upregulated in the fat body and ovary but was downregulated in the thorax after blood-feeding. These expression patterns of the ILPs and InR genes suggest a possible role in regulating the nutrition metabolism during blood-feeding.

**Fig. 6:**
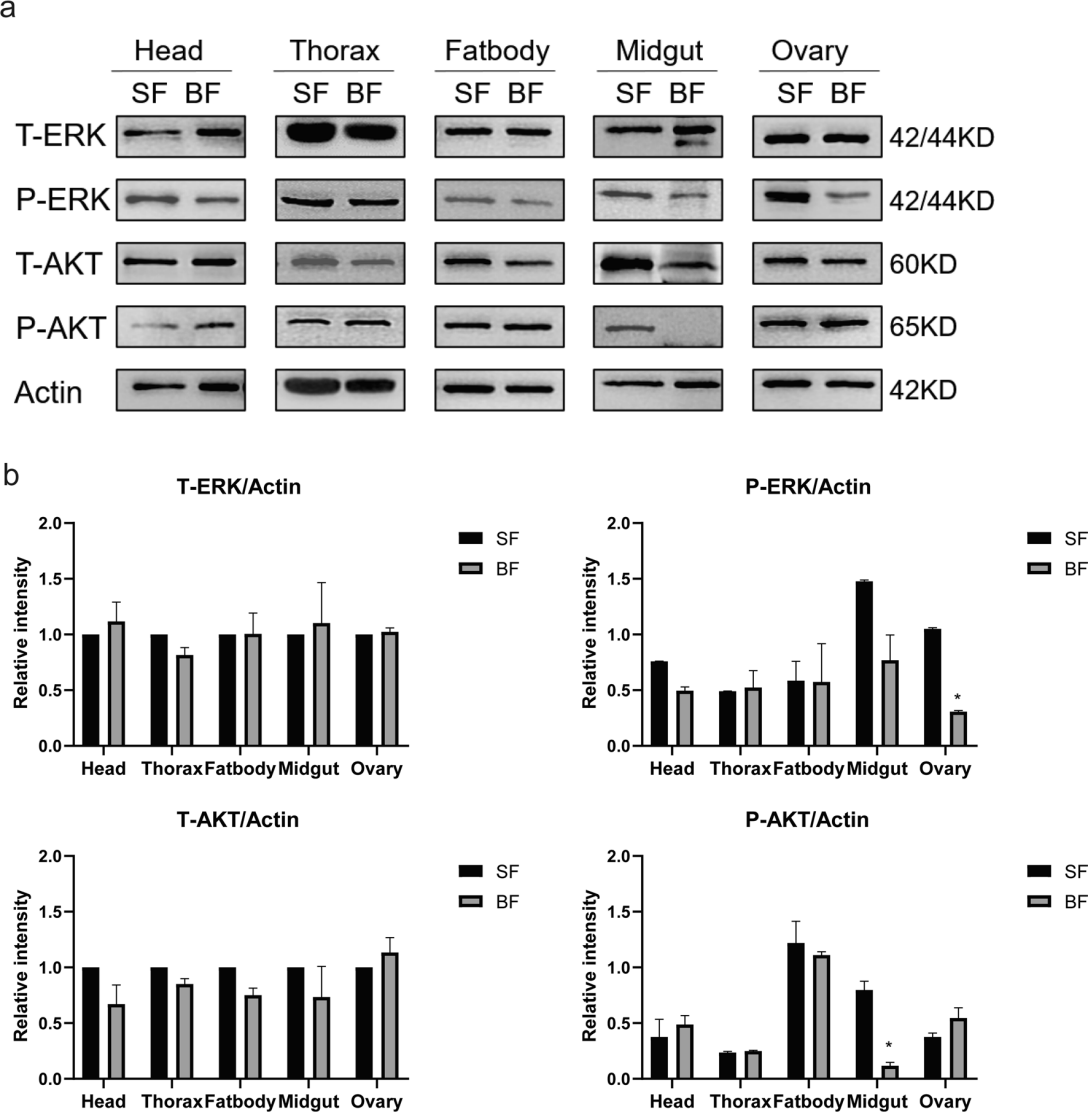
Tissue expression of ERK and AKT proteins in the female mosquito. **a** The proteins of female adults from sugar-feeding (SF) and blood-feeding (BF) groups were extracted and subjected to SDS-PAGE. Total and phosphorylation of ERK and AKT proteins were detected by Western blotting analysis. The ratios of ERK to actin were determined by using band density analysis. **b** Gray value analysis of total ERK/actin (T-ERK/actin), phosphorylated-ERK/actin (P-ERK/actin), total AKT/actin (T-AKT/actin) and phosphorylated-AKT/actin (P-AKT/actin). Data are presented as mean ± SEM from three independent experiments, *P < 0.05

Next, we examined ERK and AKT protein levels in female adult tissues after blood-feeding. Blood meals did not influence the T-ERK protein levels in different tissues of female adults, but significantly inhibited the P-ERK protein levels in the ovary (Fig. 6a and b). Similar to the T-ERK, blood meals did not change the T-AKT protein levels in the different tissues of females: head, thorax, fat body, midgut and ovary. However, we observed that the P-AKT protein level was rarely detected in blood-feeding midguts compared to those in the sugar-feeding midguts (Fig. 6a and b). The results showed that blood meals regulate the IS pathway, which exhibited variety in the female midgut and ovary.

### Effect of dsRNA-induced AalbInR knockdown on the mosquito development

**Fig. 7:**
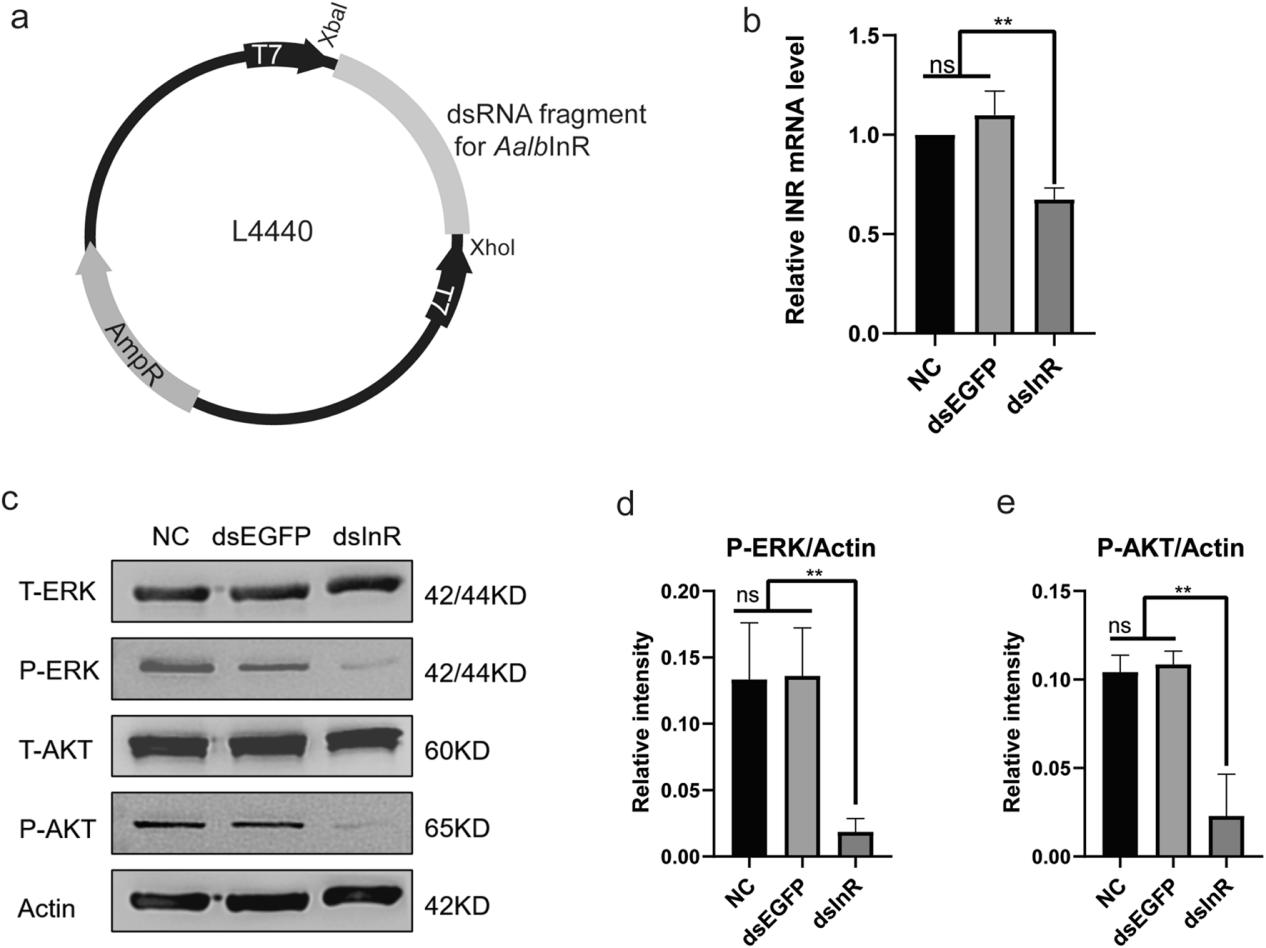
Knockdown of InR by feeding the larvae with bacterial-expressed double-stranded RNA (dsRNA). **a** Schematic diagram of the bacterial vector (L4440) construction. The target gene fragment was inserted in the multiple-cloning site (Xba I/Xho I) between two T7 promoter regions. The recombinant L4440 plasmids were then transformed into the RNase III-deficient *E. coli* strain HT115 (DE3). IPTG induces dsRNA transcription mediated by T7 promoter. **b** The bacteria producing InR dsRNA (dsInR) were fed to mosquito larvae (3rd instar) for 48 h; the transcription level of InR was analyzed by RT-qPCR. EGFP dsRNA-fed larvae (dsEGFP) were taken as the dsRNA control, and the larvae fed with no bacterial LB mixture were taken as the negative control (NC). **c** Total and phosphorylation of ERK and AKT proteins were detected by Western blotting analysis. **d** Gray value analysis of phosphorylated-ERK/actin (P-ERK/actin). **e** Gray value analysis of phosphorylated-AKT/actin (P-AKT/actin). Data are presented as mean ± SEM from three independent experiments; ns represents no significance, **P < 0.01

Mosquitoes produce 5–8 ILPs and only one known InR [6, 16, 24]. To investigate the effect of IS pathway on *Ae. albopictus* mosquito development, we adopt the strategy of microbial-base dsRNA interference to knock down InR (Fig. 7a). The transformed *E. coli* expressing InR dsRNA (dsInR) were confirmed by PCR (Additional file 3: Fig. S3) and then fed to L3 larvae by oral administration, and the recombinant *E. coli*-producing EGFP dsRNA (dsEGFP) was taken as the dsRNA control. As shown in Fig. 7b, feeding larvae with dsInR significantly reduced the transcription levels of InR gene (~ 40%) compared to the dsEGFP-fed and the NC groups. Next, we examine the expression levels of ERK and AKT proteins. Knockdown of InR did not change the total protein levels for both ERK and AKT in the larvae, but phosphorylated levels of ERK and AKT proteins were significantly decreased (Fig. 7c–e). This demonstrated that knockdown of InR successfully blocks the ERK/AKT cascades in the IS pathway by feeding the larvae with *E. coli*-producing dsRNAs.

**Fig. 8:**
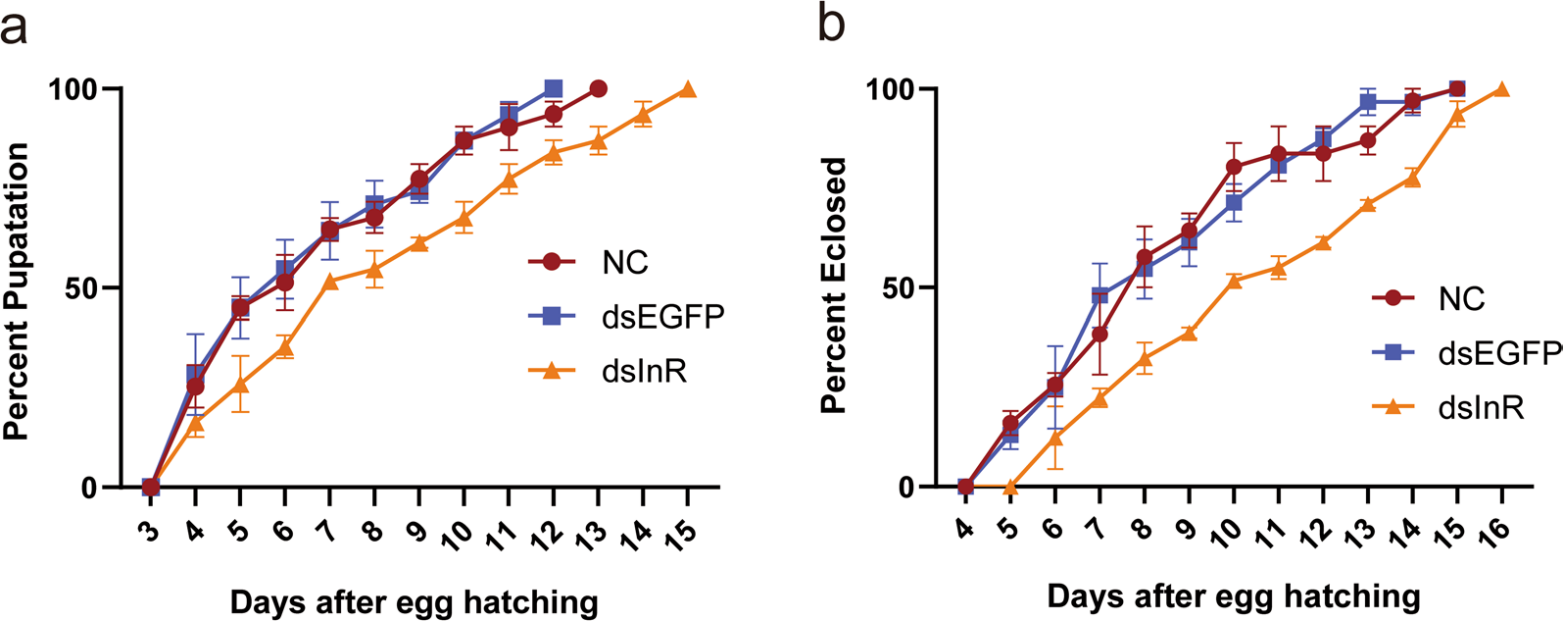
Maturation time of mosquito larvae after feeding dsRNA. **a** The percentage of pupation. **b** The percentage of eclosion. Each treatment with 10–20 individuals was analyzed. The log-rank test was used for the pupation curves and eclosion curves (*P* < 0.001). Deep red line indicates NC group. Blue line indicates dsEGFP group. Yellow line indicates dsInR group

To investigate the effect of InR knockdown on the development of *Ae. albopictus* mosquito, three groups of larvae, the dsInR group, dsEGFP group and NC group, were raised and maintained under identical conditions at all stages. The ratioes of pupation and eclosion were recorded daily after feeding larvae with dsRNA to measure their developmental conditions. We observed that silence of InR significantly reduces the ratio of pupation after feeding *E. coli*-producing dsRNAs in 5 days compared with dsEGFP treatment, and the inhibition effect on the pupation can last for > 10 days (Fig. 8a). No significant changes were found between the dsEGFP group and the NC group. Meanwhile, the eclosion times of mosquitoes after feeding InR dsRNA were also delayed compared to those in the dsEGFP group and the NC group (Fig. 8b).

**Fig. 9:**
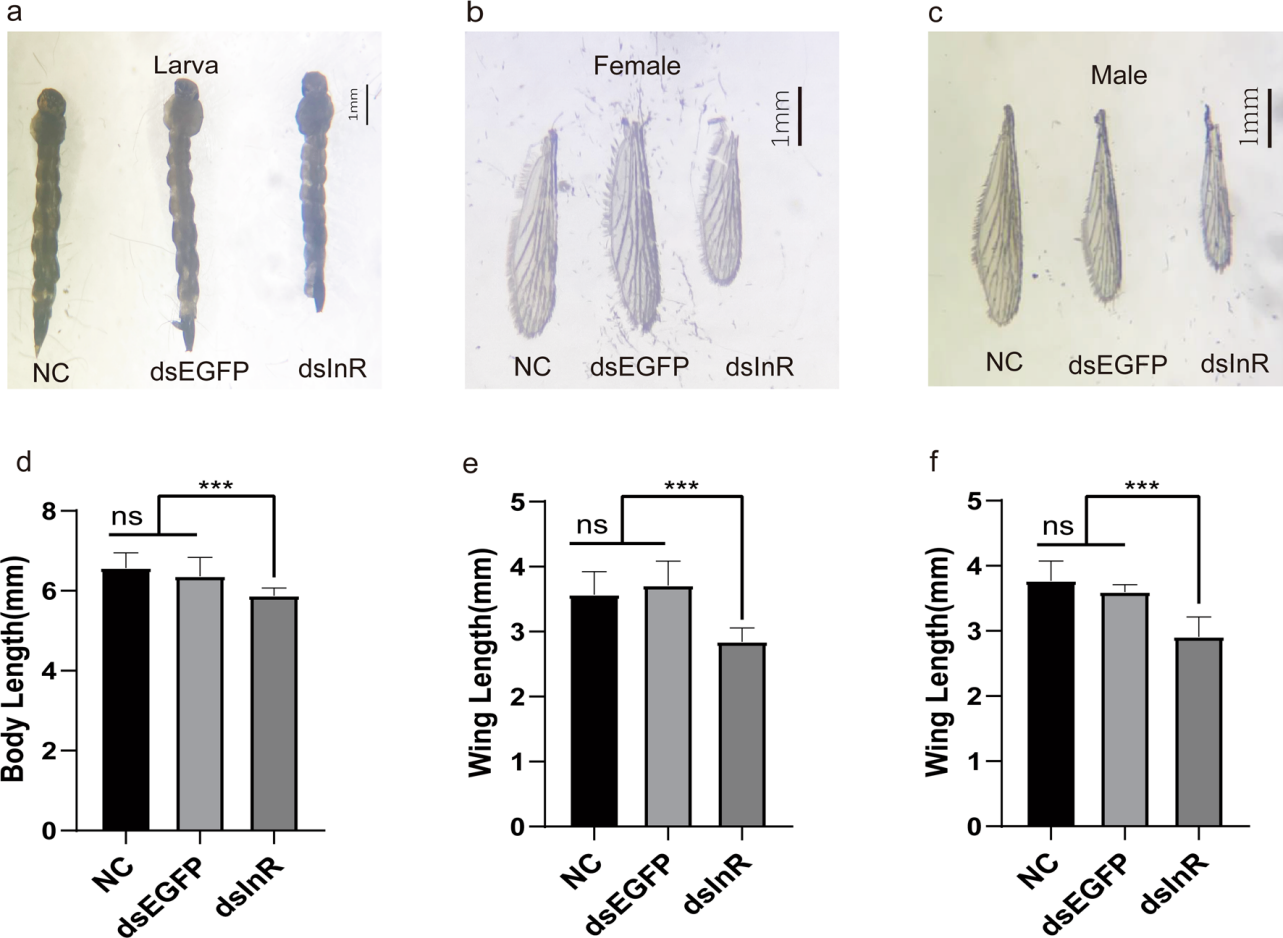
Body size and wing size variations in mosquitoes after feeding dsRNA. **a** Comparison of body length of larvae after feeding with InR dsRNA (dsInR), EGFP dsRNA (dsEGFP) and no dsRNA (NC), respectively. **b** Comparison of wing length of female adult after feeding with InR dsRNA (dsInR), EGFP dsRNA (dsEGFP) and no dsRNA (NC), respectively. **c** Comparison of wing length of female adult after feeding with InR dsRNA (dsInR), EGFP dsRNA (dsEGFP) and no dsRNA (NC), respectively. Scale bar, 1 mm. All adults (*n* = 10–20) were used for measurement. ***P < 0.005

We also evaluated the impact of InR silence on the body size of larvae and adults. Body sizes of the last-instar larva, female and male adults were recorded. The larvae in InR dsRNA group exhibited a much smaller body size while larvae in the dsEGFP group exhibited a similar body size to those in the NC group (Fig. 9a and d). The influence on body size could last until the adult stage (Additional file 4: Fig. S4). Meanwhile, the wing length of adult mosquitoes (both male and female) was measured to ascertain whether wing size is attuned to the body sizes. We confirmed that the wing length of adults (both male and female) in the dsInR group was shorter than those in the dsEGFP group, and there was no difference between the dsEGFP and NC groups (Fig. 9b, c, e and f). These results indicated that the knockdown of InR in larvae makes the body sizes smaller when developing into adults.

## Discussion

*Aedes albopictus* is an important vector for transmitting dengue, chikungunya and Zika viruses and is quickly spreading worldwide currently [22]. Mosquitoes need to endure metamorphosis before the female adults suck the human blood and transmit vector-borne diseases [28]. The IS pathway plays a critical role in regulating the metabolism, growth, reproduction and development in a variety of insect species [2, 29, 30]. In this study, we characterized the molecular and expression characteristic of IS pathway-related genes, ILPs, InR and AKT/ERK, in the lifespan and different tissues of *Ae. albopictus*. Additionally, we investigated the potential role of IS pathway in mosquito development.

We identified seven putative ILP genes (ILP1-7) in the *Ae. albopictus* assembly. The genome of the related mosquito *Ae. aegypti* contains eight ILP genes [5]. However, no gene encoding ILP8 has been identified in *Ae. albopictus*. Seven ILP genes were also found in the genome of *An. stephensi* but only five unique ILPs because of two duplicated ILP genes that encode identical B- and A-peptides [6]. Seven ILP genes of *Ae. albopictus* were conserved in the phylogenetic tree with ILPs from other insects, especially for *Ae. aegypti* ILPs [5]. Amino acid sequence alignments demonstrated that seven ILPs have a similar propeptide structure to the insulin superfamily including the SP, B-chain, C-chain and A-peptides, which was common in both invertebrate and vertebrate ILPs [1, 24]. Domain analyses revealed the amino acids between ILPs family members of *Aedes* mosquitoes are conserved key residues necessary for appropriate processing and tertiary structure. This evolutionary conservation suggests a critical physiological role for insect ILPs [24].

Insects undergo distinct developmental stages, and these stages possess very distinct nutrient acquisition and metabolism conditions [1, 2]. In *Drosophila*, ILPs play an important role in regulating feeding preference and metabolism [2]. Transcriptional profiles of ILP1-7 revealed low expression levels for all ILPs in the egg stage, which is consistent with the expression pattern of their orthologue ILPs in *Ae. aegypti* mosquito [5]. It is suggested that ILP genes may not be constitutively active in embryonic development [24]. When developing into the larval stage, the mRNA transcription of seven ILPs is increased, indicating that the ILP1-7 are involved in supporting the metabolic needs during development [17]. Meanwhile, we observed the expression levels of four ILP (ILP3, -5, -6 and -7) genes were higher in male adults, while ILP2 expression was much higher in female adults, especially in the ovary tissues. Male mosquitoes solely obtain carbohydrates from their diets; however, female mosquitoes need to change their diet from carbohydrate-rich nectar to vertebrate blood for ovary development [31, 32]. The different expression of ILPs between male and female mosquitoes may reflect their distinct functions in regulating nutrient acquisition [24].

Blood-feeding is an important physiological activity for female mosquitoes [32]. Blood meals trigger ILPs released from specific cells or organs into circulation to digest protein and lipids as well as stimulate egg maturation [6, 32]. The nervous system is considered the primary source of insect ILPs [1, 4]. Blood-feeding induces ILP2, -3, -6 and -7 mRNA expression upregulationed in the female brain. ILP3 is the most abundant ILP in the brain of *Ae. albopictus*. The release of ILP3 from the brain of *Ae. aegypti* after blood-feeding regulates the enzymes that digest the blood meal and yolk protein expression by the fat body to stimulate the proliferation of hemocytes [32]. Insect midgut and fat body are also considered important sources of ILPs [5, 6, 33]. We observed ILP2 and ILP6 expressions were upregulated in the midgut of female adults after blood-feeding. In *Drosophila*, ILP2 is the most closely related to mammalian insulin, and ILP6 is characterized as the putative IGF-like ILP [4]. The insect fat body is an analogue of the vertebrate liver and adipose tissue [12, 33]. Overexpression of ILP6 in the *Drosophila* adult fat body extends lifespan and increases longevity-associated metabolic phenotypes [33]. However, *Drosophila* fat body ILP6 expression represses other ILP production and secretion in the brain [33]. In *Ae. aegypti*, blood-feeding boosts serotonin concentration and elevates the serotonin receptor Aa5HT2B transcript level, which modulates the expression of ILP2, -4, -5 and -6 in the fat body [29].

IS pathway is activated by the binding of ILPs and InR and promotes the phosphorylation level of ERK and AKT proteins [34]. Although a variant number of ILPs has been identified in mosquitoes, only a single known InR was reported for a long time in both *Aedes* and *Culex* species [16, 19]. The InR from *Ae. aegypti* and *Drosophila* are similar to mammalian InR/IGFRs [16, 34]. Complete removal of InR in *Drosophila* throughout development causes early larval lethality [35]. The deficiency of InR reduces lipid synthesis and reproductive function in the insect *Rhodnius prolixus*, a vector of Chagas disease [18]. We found that InR expression is increased in the post-embryonic development stages. Application of bacteria expressing dsRNA to knockdown InR in the larval stage led to the delay of development and much smaller body size in the adults, which may be related to the blockade of ERK and AKT cascades. Similarly, RNAi against the IS pathway genes of *Maruca vitrata* can interfere with larval growth, leading to small pupae or significant larval mortality [36]. This indicated that the bacteria expressing dsRNA specific to IS components can be used to develop dsRNA insecticide [37].

## Conclusion

Here we present expression analyses of the ILP gene family and the functional study of InR in *Ae. albopictus* mosquito development. Our study confirmed IS pathway genes ILP1-7, InR and ERK/AKT in *Ae. albopictus* genome assembly. Sequence homology suggested conservation between ILP and other insects' ILPs, and the presence of insulin family domains supports ILPs as a factor in regulating the development and metabolism of *Ae. albopictus*. Expression characteristics revealed complexities of IS pathway genes between stages and sexes and suggest that the IS pathway of female adult mosquitoes is regulated by blood-feeding. In addition, it demonstrated that transformed *E. coli* expressing dsRNA specific to InR has significant inhibition activity with oral application, which can be used in the development of novel control tools for *Ae. albopictus* mosquitoes.

## Supplementary Information

**Additional file 1: Figure S1.** Phylogenetic tree of the known dipteran insulin receptor.

**Additional file 2: Figure S2.** Expression of ILPs and InR in different tissues of female adults.

**Additional file 3: Figure S3.** PCR identification of the recombinant bacterial vector L4440-dsInR and L4440-dsEGFP.

**Additional file 4: Figure S4.** The body size of the female and male adults after feeding dsRNA.

**Additional file 5: Table S1.** The accession number of dipteran ILP and InR genes in NCBI. **Table S2.** The primer sequences for RT-qPCR analysis and dsRNA amplification.

## Data Availability

Most data generated or analyzed during this study are included in this published article and its additional information files. Any related data are available from the corresponding author upon reasonable request.

## Abbreviations

*Ae. albopictus*: *Aedes albopictus*
*Ae. aegypti*: *Aedes aegypti*
*An. gambiae*: *Anopheles gambiae*
*D. melanogaster*: *Drosophila melanogaster*
*B.mori*: *Bombyx mori*
IS: Insulin-like signaling
ILPs: Insulin-like peptides
InR: Insulin receptor
IGFPs: Insulin growth factor peptides
IRSs: Insulin receptor substrates
ERK: Extracellular regulated protein kinases
AKT: Protein kinase B
SP: The signal peptides
RT-qPCR: Real-time quantitative PCR
IPTG: Isopropyl-β-d-1-thiogalactopyranoside
T-ERK: Total ERK
T-AKT: Total AKT
P-ERK: Phosphorylated-ERK
P-AKT: Phosphorylated-AKT
