# Supplementary figures and images for: Molecular and expression characterization of insulin-like signaling in development and metabolism of Aedes albopictus
Source: Parasit Vectors. 2023 Apr 18;16:134. doi: 10.1186/s13071-023-05747-8 (PMC10111782; doi:10.1186/s13071-023-05747-8)

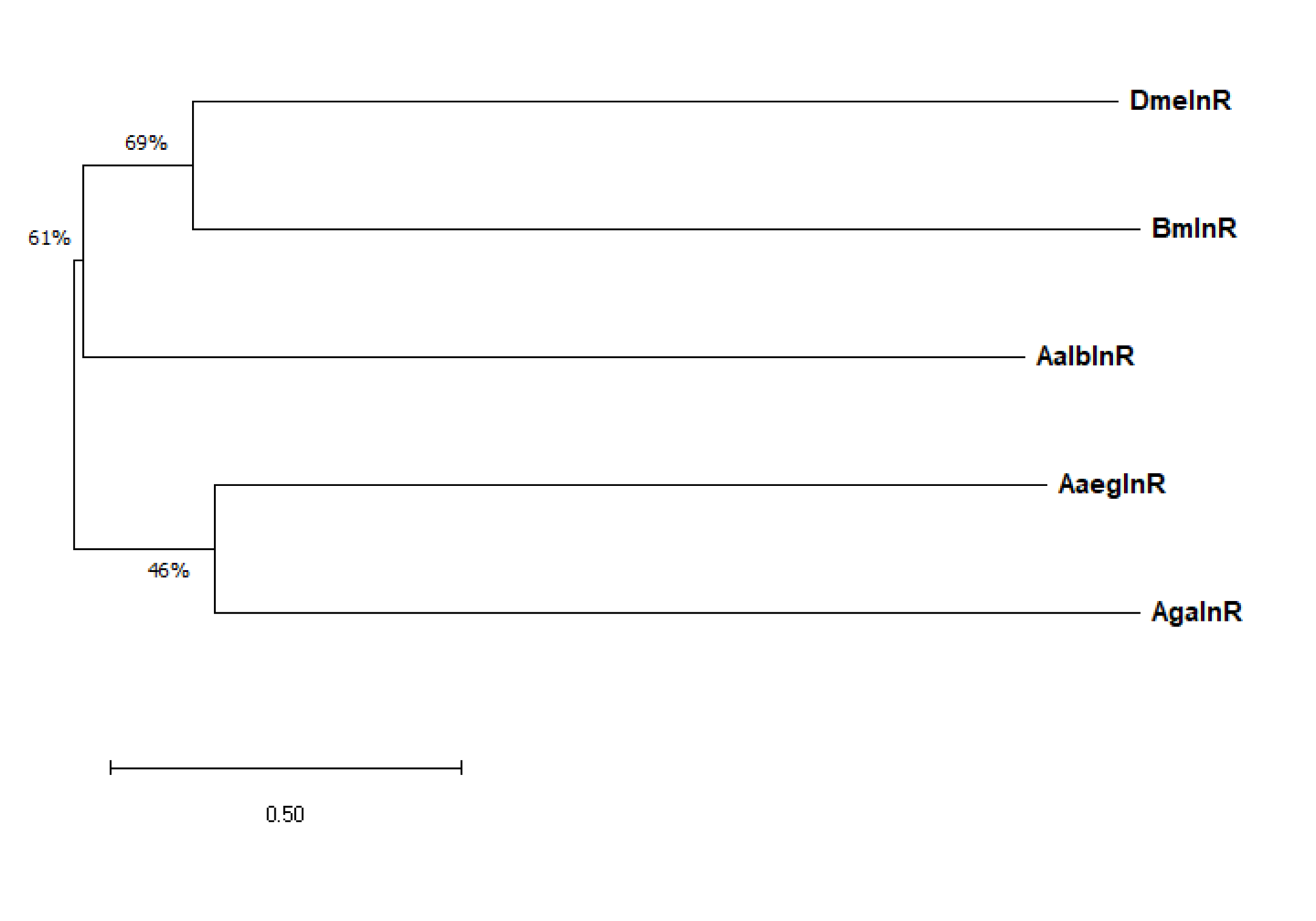

Supplement: Supplementary file 1 — Additional file 1: Figure S1. Phylogenetic tree of the known dipteran insulin receptor. [file 13071_2023_5747_MOESM1_ESM.tif]

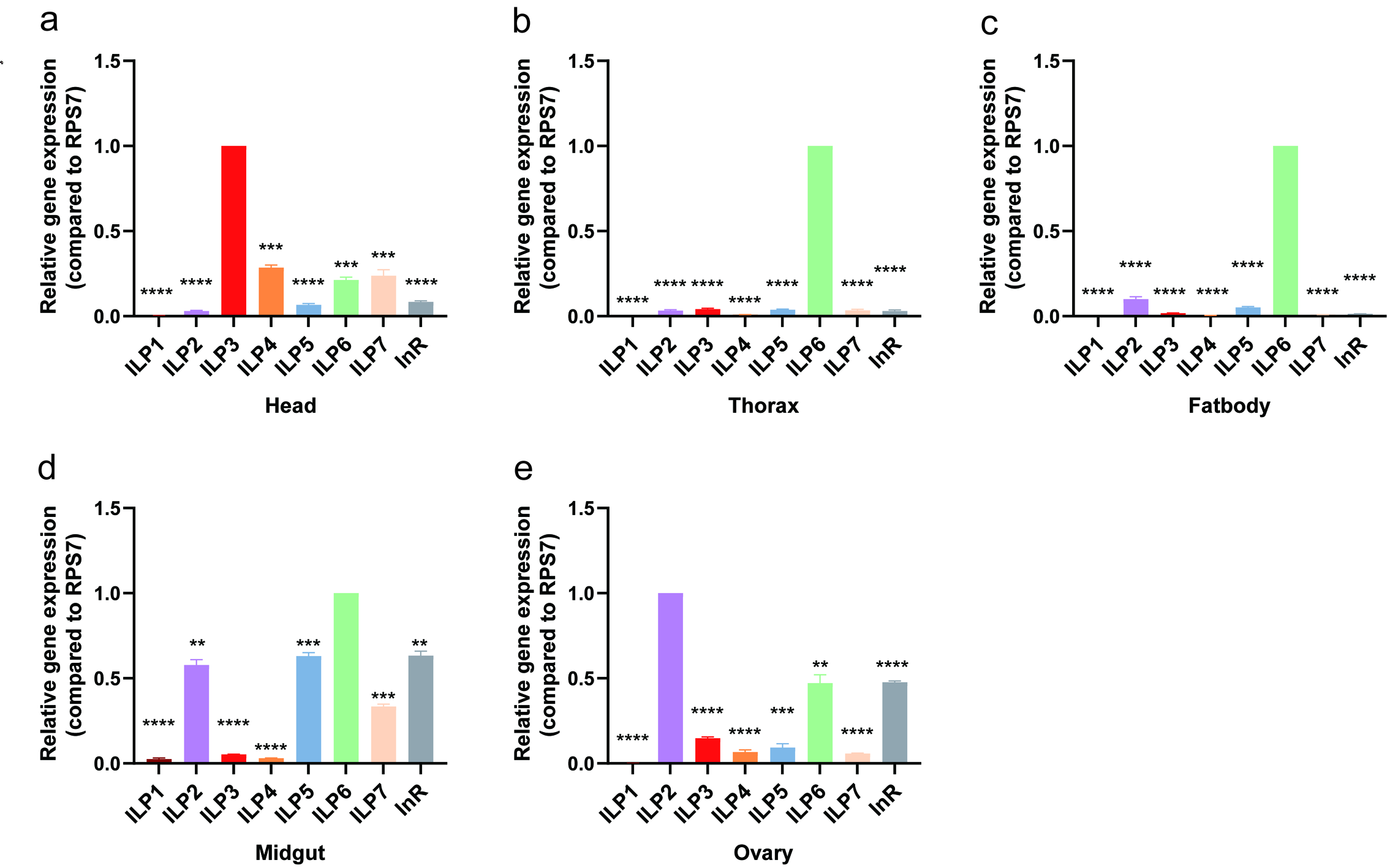

Supplement: Supplementary file 2 — Additional file 2: Figure S2. Expression of ILPs and InR in different tissues of female adults. [file 13071_2023_5747_MOESM2_ESM.tif]

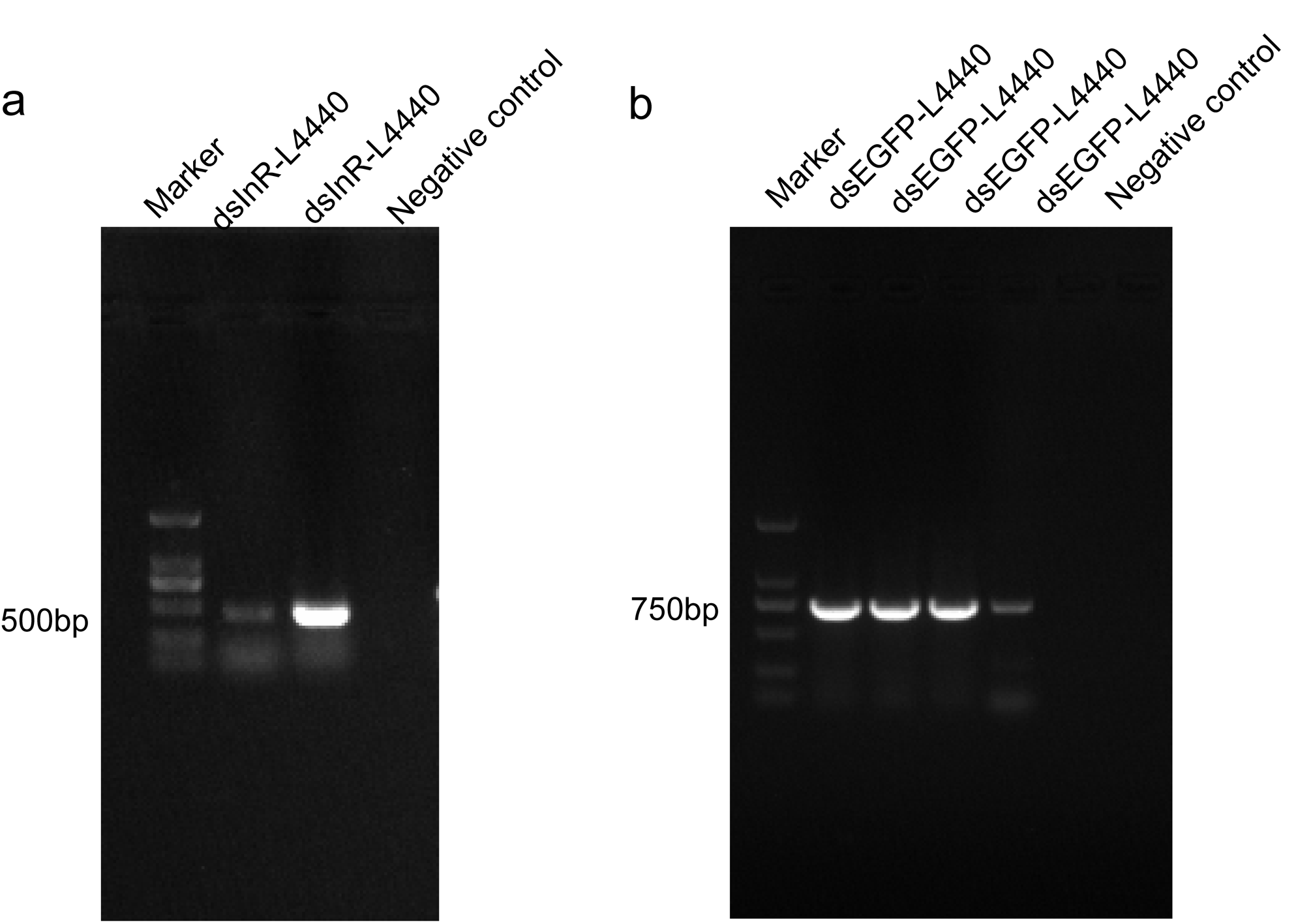

Supplement: Supplementary file 3 — Additional file 3: Figure S3. PCR identification of the recombinant bacterial vector L4440-dsInR and L4440-dsEGFP. [file 13071_2023_5747_MOESM3_ESM.tif]

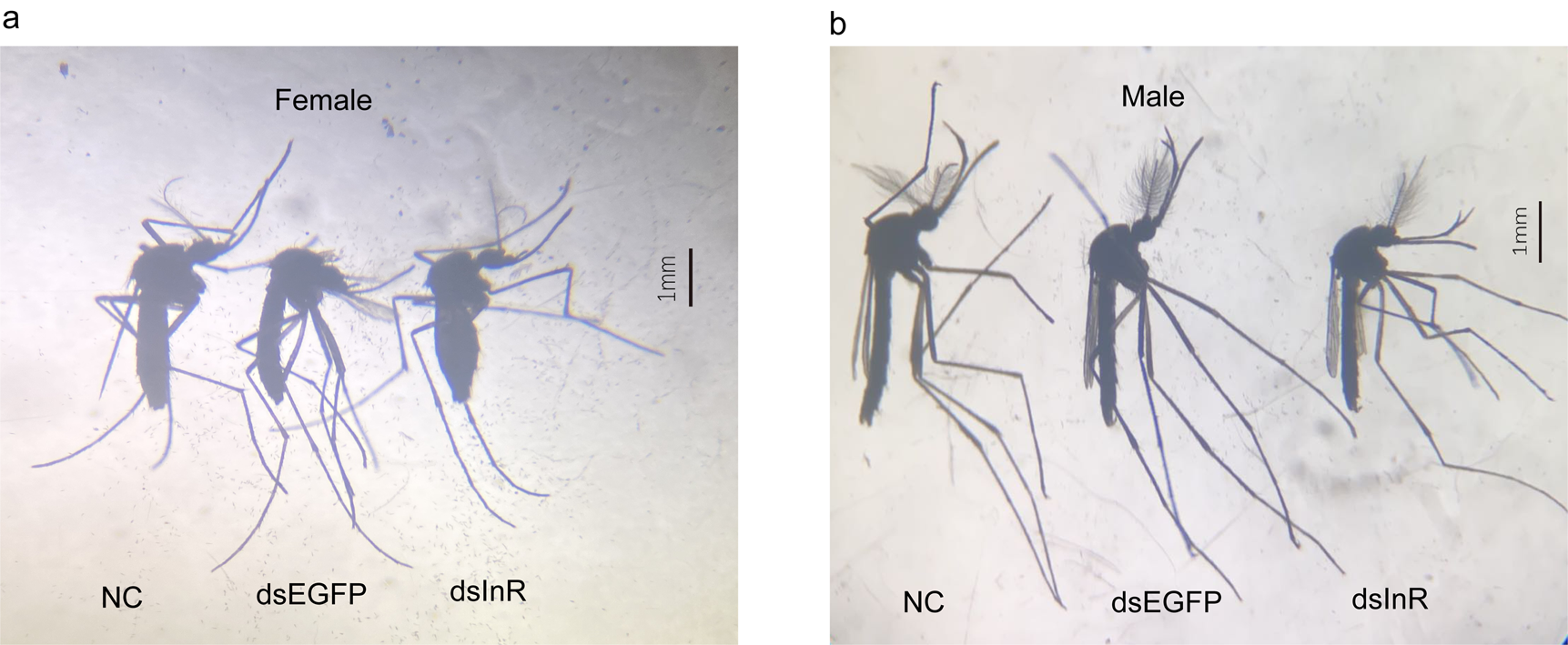

Supplement: Supplementary file 4 — Additional file 4: Figure S4. The body size of the female and male adults after feeding dsRNA. [file 13071_2023_5747_MOESM4_ESM.tif]
