# Supplementary material for: Molecular and expression characterization of insulin-like signaling in development and metabolism of Aedes albopictus
Source: Parasit Vectors. 2023 Apr 18;16:134. doi: 10.1186/s13071-023-05747-8 (PMC10111782; doi:10.1186/s13071-023-05747-8)
Supplement: Supplementary file 5 — Additional file 5: Table S1. The accession number of dipteran ILP and InR genes in NCBI. Table S2. The primer sequences for RT-qPCR analysis and dsRNA amplification. [file 13071_2023_5747_MOESM5_ESM.docx]

**Additional file**

**Supplemental Figures**

**Fig. S1** Phylogenetic tree of the known dipteran insulin receptor. The unrooted tree of insulin receptor (InR) generated with ClustalW alignment and neighbor-joining analysis. *Ae. albopictus* (Aalb), *Ae.aegypti* (Aaeg), *An.gambiae* (Aga) and *D. melanogaster* (Dme), *Bombyx mori* (Bm).

**Fig. S2** Expression of ILPs and InR in different tissues of female adults. Relative expression levels of ILPs and InR genes in the head, thorax, fat body, midgut and ovary of female mosquitoes. RNA levels between samples were normalized to the RPS7 gene using 2^−ΔΔCt^ method. Data represent three biological replicates with 20 individuals in each and are shown as mean ± SEM , ** *p* < 0.01, *** *p* < 0.005, **** *p* < 0.001.

**Fig. S3** PCR identification of the recombinant bacterial vector L4440-dsInR and L4440-dsEGFP. The InR gene fragmentand EGFP genewere inserted in the multiple-cloning site between two T7 promoter regions, respectively. The recombinant L4440 plasmids were then transformed into the RNase III-deficient *E. coli* strain HT115 (DE3). **a** the InR gene fragment (411bp) were analyzed by PCR using the specific primers. **b** the EGFP gene (717bp) were analyzed by PCR using the specific primers.

**Fig. S4** The body size of the female and male adults after feeding dsRNA. **a** Comparison of body length of female adults after feeding with InR dsRNA (dsInR), EGFP dsRNA (dsEGFP) and no dsRNA (NC), respectively. **b** Comparison of wing length of male adults after feeding with InR dsRNA (dsInR), EGFP dsRNA (dsEGFP) and no dsRNA (NC), respectively.

**Supplemental Tables**

**Table S1.** The accession number of dipteran ILP and InR genes in NCBI

| **Organism** | **Gene name** | **NCBI Accession Number** |
| --- | --- | --- |
| ***D. melanogaster*** | DmeILP1  DmeILP2  DmeILP3  DmeILP4  DmeILP5  DmeILP6  DmeILP7  DmeILP8  DmeInR | NM_140102.2  NM_079288.3  NM_140103.3  NM_140104.3  NM_206315.2  NM_130644.4  NM_130714.2  NM_140692.3  NM_079712.6 |
| ***An. gambiae*** | AgaILP1  AgaILP2  AgaILP3  AgaILP4  AgaILP5  AgaILP6  AgaILP7  AgaInR | XM_558134.2  XM_314564.3  XM_001237578.1  XM_314565.3  XM_001231147.2  XM_314567.3  XM_558137.2  XM_320130.3 |
| ***Ae. aegypti*** | AaegILP1  AaegILP2  AaegILP3  AaegILP4  AaegILP5  AaegILP6A  AaegILP6B  AaegILP7  AaegILP8  AaegInR | DQ_845750.1  DQ_845752.2  DQ_845751.2  DQ_845753.1  DQ_845758.1  DQ_845755.1  DQ_845756.1  DQ_845757.1  DQ_845754.1  XM_001651160.2 |
| ***Ae. albopictus*** | AalbILP1  AalbILP2  AalbILP3  AalbILP4  AalbILP5  AalbILP6 | XM_019675899.3  XM_019675941.3  XM_029875704.1  XM_029876365.1  XM_019672341.2  XM_019707062.2 |
|  | AalbILP7  AalbInR | XM_019675900.2  XM_029853782.1 |
| ***B. mori*** | BmInR | XM_019675900.2 |

Note: Dme: *D. melanogaster*; Aga: *An.gambiae*; Aaeg: *Ae.aegypti*; Aalb: *Ae. Albopictus*; Bm: *B. mori.*

**Table S2.**The primer sequences for RT-qPCR analysis and dsRNA amplification.

| **Target genes** | **Primers** |
| --- | --- |
| qAalbILP1  qAalbILP2  qAalbILP3  qAalbILP4  qAalbILP5  qAalbILP6  qAalbILP7  qAalbInR  qRPS7  dsInR  dsEGFP | F: 5’- TTGCTGCTGATAGCGTTGGTC -3’  R: 5’-GTAGGATTCGTCGTTGGTGGC- 3’  F: 5’- AAACGCTGGCCCTGTTATGC-3’  R: 5’ -AAACCGTGGCTATTGCTGTCC-3’  F: 5’-TATGGTCGGAACAGTAGTACACGC-3’  R: 5’- AGTCTTTGTCGTAGTCGCTAATGGA-3’  F: 5’ -AACGCTGAAGGACAAGCCAAAC-3’  R: 5’-ACGGCAACATTCCTCCACGATA-3’  F: 5’- GGGTCCTACTGATGGATACGGT-3’  R: 5’- GCCAAACTTTTTCCCAATCG-3’  F: 5’ -CGCATCAAGCGAGGAATAGTC-3’  R: 5’ -GCGGTTGTTGGTGGAGTAGTG -3’  F: 5’- GTTTCCGAACTTTCCACCA- 3’  R: 5’ -GCCATCCACCACCACTTTA-3’  F: 5’- CCCAGAAGGAAGGAGCCGATGA-3’  R: 5’ -GGGTACTTGCCCACAAACTTCACA- 3’  F: 5’- GAAGTTGTCGGAAAGCGTATGC-3’  R: 5’ -TTCAATGGTGGTCTGCTGGTTC-3’  F: 5’-GCTCTAGAGGTTCGCCTGGTGGACATTGAG-3’  R: 5’- CCCTCGAGATCGTGGTGCCCATCGAGTCAT-3’  F: 5’- GCTCTAGAATGGTGAGCAAGGGCGA-3’  R: 5’-CCGCTCGAGTTACTTGTACAGCTCGTCC -3’ |

Note: F: Forward primer; R: Reverse primer.
